# Affordability and Serving Accuracy of a Publicly Available DASH Meal Plan for Women Using SNAP Benefits

**DOI:** 10.3390/nu18091358

**Published:** 2026-04-25

**Authors:** Kendra OoNorasak, Mansura Shahad Bawa, Nadia A. Sesay, Emma Smith, Taylor Emerson, Jayden Brewer, Brandi M. White

**Affiliations:** 1 Department of Health and Clinical Sciences, College of Health Sciences, University of Kentucky, Lexington, KY 40536, USA; msh380@uky.edu (M.S.B.); nadia.sesay@uky.edu (N.A.S.); emma.smith2248@uky.edu (E.S.); jebr265@uky.edu (J.B.); brandi.white50@uky.edu (B.M.W.); 2 College of Communication, University of Kentucky, Lexington, KY 40506, USA

**Keywords:** DASH, food cost, grocery, serving, thrifty food plan, SNAP, heart health, cardiovascular, woman

## Abstract

**Background/Objectives**: This regional case study evaluated the affordability and serving accuracy of a publicly available one-week DASH meal plan for single-woman households using Supplemental Nutrition Assistance Program (SNAP) benefits in central Kentucky. **Methods**: For each food item in the one-week plan, total grocery costs and per-serving costs were calculated using January 2025 prices from two national grocery chains commonly patronized in an urban area in central Kentucky. Calculated costs were compared to the average weekly food cost for women aged 20–50 years in a single household based on the Thrifty Food Plan (TFP). Servings for food groups and categories were calculated using MyPlate and American Heart Association guidelines to compare with those reported in the one-week plan. **Results**: The total grocery cost was $262.17, including staple foods expected to last more than a week. The adjusted per-serving cost of $82.90 was 21.19% higher than the average weekly food cost based on the January 2025 TFP. All food groups and categories except dairy showed differences of at least one serving between our calculations and the one-week plan. **Conclusions**: Findings from this case study on grocery costs suggest that the one-week plan may pose affordability challenges in this regional context and continued evaluation of whether SNAP benefit allotments based on TFP adequately reflect regional food cost variations may be warranted. Discrepancies in total servings highlight the need to improve the accuracy of publicly available DASH resources and to review these materials for consistency and accuracy.

## 1. Introduction

Cardiovascular disease (CVD) remains a leading cause of death among women globally, accounting for 35% of all deaths [1]. Hypertension is the strongest risk factor for the development of stroke and other CVDs [2]. In the United States, almost half of all adults have hypertension, also known as high blood pressure [3]. The highest prevalence rates are noted among adults with low socioeconomic status and women [4,5,6]. Women represent a particularly vulnerable population, as they are often underdiagnosed and undertreated, which contributes to worse health outcomes compared to men [4,5,6].

One important factor affecting heart health among populations with low socioeconomic status (SES) is food insecurity, defined as limited access to affordable, nutritious food [7]. Individuals experiencing food insecurity often rely on foods with poor nutritional value, such as excessive intake of sodium, added sugars, and unhealthy fats, while consuming limited amounts of fruits, vegetables, whole grains, fiber, legumes, fish, and nuts [8]. Such dietary patterns are common risk factors for CVD; thus, food insecurity is commonly associated with poor dietary quality and an increased risk of diet-related health conditions, including CVD [7].

Evidence demonstrates that nutrition plays a critical role in cardiovascular health. A prospective cohort study found that consuming at least 4 servings of fruits and vegetables per week significantly reduced the risk of hypertension when compared with consuming fewer than 1 serving per month [9]. The DASH (Dietary Approaches to Stop Hypertension) eating plan is an evidence-based nutrition intervention recommended by the National Heart, Lung, and Blood Institute (NHLBI) [10]. This eating plan emphasizes fruits, vegetables, lean meats, whole grains, low-fat dairy, legumes, nuts, and seeds as sources of key macronutrients- and micronutrients, while recommending a limited intake of sodium, added sugars, and saturated fats [10]. Daily recommendations for DASH include 4–5 servings of fruits and vegetables, 6–7 servings of grains, and up to 6 servings of lean proteins [10]. The DASH eating plan complements medication for various cardiovascular conditions, as adoption and adherence to the DASH plan can significantly reduce overall hypertension risk and insulin resistance, with lowered blood pressure, waist circumference, cholesterol, and triglycerides [11,12].

While the DASH eating plan offers strong evidence for cardiovascular health promotion, food insecurity and economic barriers can make its adoption and adherence challenging, particularly among low-SES populations. The U.S. Department of Agriculture (USDA)’s Supplemental Nutrition Assistance Program (SNAP), formerly known as the Food Stamp Program, provides monetary assistance for food expenditures. The amount of monetary support for eligible individuals is calculated based on the Thrifty Food Plan (TFP) [13]. However, the TFP has notable limitations, including oversimplifying dietary needs for certain populations (e.g., pregnant and lactating women) and failing to account for components of the Dietary Guidelines for Americans [14,15]. Particularly, women living alone and single-woman households with children experience food insecurity at disproportionately higher rates than men and other households in the U.S. [16,17]. This can impede women’s ability to adopt and adhere to the DASH plan, even though women are at high risk for CVD.

Additionally, research highlights an alarming mismatch between SNAP benefit allotments and the cost of following a nutritious diet [14,18]. A study estimated that a nutritious diet for a low-income family of four costs $692 per month, which far exceeded the maximum SNAP benefit of $242, calculated using the TFP [14]. This cost gap illustrates the limitations of existing food assistance programs in supporting nutritionally adequate diets that may promote heart health. This gap is concerning because healthy dietary patterns promoting cardiovascular health are associated with higher food expenditures, and the DASH plan generally recommends the consumption of fresh fruits, vegetables, nuts, seeds, whole grains, lean proteins, and low-sodium foods, which can incur high food costs [14,18].

Despite NHLBI’s widespread promotion of the DASH plan, no study has rigorously examined its economic feasibility for SNAP recipients. This gap in current literature is particularly relevant as household structures shift in the U.S., where more women are living alone. In 2022, 15.70% of all households in the U.S. were represented by a woman living alone, an increase from 11.50% in 1970 [19]. Moreover, single-women households face high levels of food insecurity and experience CVD disparities [1,5]. However, little is known about whether women living alone and receiving SNAP benefits can follow DASH dietary guidelines.

To our knowledge, this is the first study that addresses the gap by examining the affordability and serving accuracy of the NHLBI’s publicly available one-week DASH plan for SNAP recipients [20]. Given the CVD disparities among women and the increasing number of single-woman households, this case study calculated the grocery and per-serving costs of the one-week DASH plan in central Kentucky and compared them with the average weekly food costs for a single woman aged 20–50 years, based on TFP. Additionally, this study assessed the serving accuracy of food groups and categories listed in the DASH plan. Findings from this study will contribute to the literature by: (1) assessing the affordability of a widely recommended heart-healthy DASH eating plan; (2) evaluating the accuracy of serving information in a publicly available DASH education resource; and (3) informing future research, policy, and practical approaches to promote cardiovascular health in similar contexts.

## 2. Materials and Methods

### 2.1. Ethical Review

This study did not involve human subjects research and thus did not need approval from an ethical review board.

### 2.2. One-Week DASH Plan

The publicly available one-week DASH plan from NHLBI provides specific ingredients and recipes for breakfast, lunch, dinner, and snacks [20]. The plan is based on 2000 calories per day and total daily servings for food groups and categories are presented [20].

### 2.3. Thrifty Food Plan (TFP)

The USDA’s January 2025 TFP average food cost report provides an average weekly food cost for women aged 20–50 years to follow a nutritious, practical, and cost-effective diet, based on the Dietary Guidelines for Americans [21]. Average food costs for a reference family of four, consisting of a man and woman aged 20–50 years with a child aged 6–8 years and a child aged 9–11 years, are used to determine the maximum SNAP allotments for all households across the U.S. [21]. For a single woman aged 20–50 years living alone in January 2025, the average weekly food cost of $57 is applied, with an additional 20% to calculate the total average weekly food cost [21]. This cost was compared with actual grocery and per-serving costs in this study.

### 2.4. Grocery Costs Calculation

Foods, condiments, and beverages listed in the one-week plan were organized by day and meal type in chronological order. Grocery costs for each food item (e.g., two tomato slices, a small apple) were obtained online in January 2025 from two large national grocery store chains commonly used by households residing in an urban city in central Kentucky. These two grocery retailers were selected because they are widely accessible, with several locations across the city, and are commonly frequented by many low-income households for their reasonable food prices in the study region. Moreover, selecting these grocery retailers with online websites enabled a convenient and careful selection of specific food items listed in the one-week meal plan, rather than using national or regional averages for limited food options. However, this study does not aim to represent national food price patterns and instead presents a regional case study from central Kentucky that may have relevance to some areas of the southern U.S. states.

Instead of individual food items, some meals included DASH-friendly recipes (e.g., chicken salad) from NHLBI’s website, which provided DASH plan resources [22]. The costs of the ingredients used in those recipes were identified online using the two grocery store websites. Data, including individual package size, serving size, and grocery cost of each food item, were recorded in a Microsoft Excel spreadsheet for calculating grocery and per-serving costs.

Additionally, the total amount needed for each repeated food item in the one-week plan (e.g., bread, milk, orange juice, and tomatoes) was noted, and large package sizes for those items were searched online. These large package sizes were used to estimate how long repeated food items would last, and grocery costs were included only for the first time the food item appeared in the meal plan. Such food items were noted for cost calculations. Any repeated food items are marked with an asterisk *, as shown in Table 1. For other food items, including staple foods (e.g., oils, cereals, condiments, seasonings, dried herbs), when only larger package sizes were available, the lowest available prices were recorded and included in the calculation, even if only a small was listed in the plan. This resulted in the inclusion of many food items that would last longer than a week. Additionally, food waste or spoilage was not included in cost calculations. For all food items, per-serving costs were calculated based on only the amount specified in the one-week plan.

When selecting food products, the population of interest, being SNAP recipients, was considered throughout the process. To standardize cost estimates and ensure reproducibility, the lowest available price across the two selected vendors and store brands was used for data entry, as they typically represent lower-cost options commonly used by low-income households. Vendors in this study include the two grocery store chains. However, regular retail prices were selected instead of sales, coupons, loyalty discounts, and clearance pricing because those temporary discounts vary by time, store, location, seasonality, and demand. This allowed for a consistent food price basis for comparison that can extend beyond the one-month period. These assumptions may result in slightly higher cost estimates depending on purchasing behaviors. In cases where the one-week plan did not specify the type of food (e.g., a small apple), the first author (KO), a registered dietitian, selected a reasonable, low-cost food item that best fit the type of meal (e.g., a small Gala apple).

#### Weight–Volume Conversions for Equivalencies

As vendors use weight units (e.g., pounds, grams, ounces) for many food products and labeling, weight-to-volume or volume-to-weight conversions were required when serving sizes in the one-week plan were provided in volume units, such as cups, tablespoons, and teaspoons. Conversions were conducted using CoolConversion, a free online tool that provides weight–volume equivalencies for a wide range of food ingredients. After determining those equivalencies, the per-serving costs of those food items were calculated. For example, this weight–volume conversion was needed for dried apricots on Day 6 of the one-week plan (see Table 1).

### 2.5. Serving Calculations Using MyPlate and American Heart Association Guidelines

Each food item in the one-week plan was assigned to the corresponding food groups and categories (e.g., fruits, vegetables, protein foods, dairy, grains, and fats/oils) according to the USDA MyPlate and the American Heart Association (AHA) guidelines [23,24]. The study team assigned the number of servings for various food groups and categories based on the amounts of individual food items listed in the plan for each day, using standardized MyPlate and AHA serving equivalencies (e.g., 1 cup of milk is 1 serving of dairy). An example of serving assignments for each food item for Day 6 of the one-week plan is provided in the Supplementary Table S1. When exact serving equivalencies were not specified, consistent assumptions based on standard serving equivalencies from MyPlate and AHA guidelines were applied with minor rounding when necessary to estimate the number of servings. These procedures were applied uniformly across all food items to ensure internal consistency. The study team then summed the servings across all food items for each food group and category to calculate the daily totals. Those totals were compared to the total servings for each food group and category indicated per day in the one-week plan. Servings of meat, fish, and poultry noted in the one-week plan were combined with those of nuts, seeds, and legumes to calculate and report total protein servings per day. All assumptions, rounding, conversions, and serving calculations in this study were reviewed and verified by the first author (KO) to ensure accuracy, consistency, and alignment with MyPlate and AHA serving guidelines.

## 3. Results

Table 1 provides an example of food items for Day 6 in the one-week plan with vendor prices for grocery costs, serving sizes, and costs per serving. The total grocery cost for all foods listed on Day 6, excluding repeated food items from previous days in the one-week plan, was $29.70. The adjusted per-serving costs to consume all the foods listed in the Day 6 plan were $9.82.

**Table 1:** Example Day 6 of the one-week DASH plan with a list of food items, vendor prices, serving sizes, and costs per serving categorized by meal types.

Meal	Food Item	Vendor Prices to Calculate Grocery Cost	Serving Size Indicated in Packaging	Serving Size in the One-Week Plan	Cost per Serving in the One-Week Plan
Breakfast	Low fat granola bar	$1.78 for 10 bars	1 bar	1 bar	$0.18
	Medium banana	$0.26 for each	N/A	1 banana	$0.26
	Fruit yogurt, fat free, sugar free	$0.72 for 5.3 oz	5.3 oz	4 oz	$0.54
	Orange juice *	$8.49 for 1 gallon/16 servings	1 cup	1 cup	$0.53
	Low fat milk *	$2.57 for 64 oz/8 servings	1 cup	1 cup	$0.32
Lunch	Turkey breast	$3.62 for 16 oz/4 servings	4 oz	3 oz	$0.68
	Whole wheat bread *	$1.97 for 20 oz/22 servings	1 slice	2 slices	$0.18
	Romaine lettuce *	$1.98 for 1 head (approximately 20 leaves)	N/A	1 leaf	$0.09
	Tomato	$0.25 for each (approximately 6 slices)	N/A	2 slices	$0.08
	Mayonnaise, low fat *	$3.40 for 30 floz/59 servings	1 tbsp	2 tsp	$0.04
	Dijon mustard *	$1.52 for 12 floz/68 servings	1 tsp	1 tbsp	$0.07
	Steamed broccoli	$0.94 for 12 oz/4 servings	¾ cup	1 cup	$0.31
	Medium orange	$0.88 for each	N/A	1 orange	$0.88
Dinner	Carrots *	$1.08 for 6 sticks (approximately 3 cups)	N/A	1 cup	$0.36
	Whole wheat roll *	$3.48 for 6 rolls	1 roll	1 roll	$0.58
	Soft tub margarine *	$1.38 for 16 oz/32 servings	1 tbsp	1 tsp	$0.01
	Small cookie	$1.48 for 3 pieces	1 cookie	1 cookie	$0.49
	Spicy baked fish with salmon, spicy seasoning, spray * and oil *	$12.83 without oil and spray cost for 4 servings in the recipe	N/A	3 oz	$2.52
	Scallion Rice	$4.46 for multiple servings ($1.56 for 5 servings in the recipe)	N/A	1 cup	$0.31
	Spinach * sauté with canola oil * and almonds, silvered, unsalted *	$1.98 for 10 oz spinach, $4.12 for 48 oz canola oil, and $5.78 for 10 oz almonds	N/A	½ cup	$0.36
Snack	Peanuts, unsalted	$2.48 for 16 oz/16 servings (approximately 0.3 oz per 1 tbsp)	1 oz	2 tbsp	$0.09
	Low fat milk *	$2.57 for 64 oz/8 servings	1 cup	1 cup	$0.32
	Dried apricots *	$5.98 for 16 oz (approximately 1 oz per 0.149 cup)	6 apricots	¼ cup	$0.62

* Indicates repeated food items that had been included in the grocery shopping list for Day 1–5; thus, were excluded from the total grocery cost calculation for Day 6.

Based on the January 2025 TFP report, an adult woman in a single household aged 20–50 years would need to spend only about $68.40 a week to follow a nutritious, practical, cost-effective diet [21]. The total cost for all food items needed for the one-week DASH plan was approximately $262.17 (see Table 2). When determining the total costs per serving for all food items, the adjusted costs for a single woman following the one-week plan totaled $82.90.

There were discrepancies in total daily servings for various food groups and categories between the study and the one-week plan. Across three days of the plan, all food groups and categories, except dairy, had at least one serving difference (see Table 3). Notably, the number of servings for fruits and vegetables in the one-week plan was higher than our calculations across all seven days. In contrast, for dairy products, there was no difference in the number of servings across the seven days. There were five days where protein servings were different, while there were three days where servings of fats/oils were different.

## 4. Discussion

This case study examined the affordability and serving accuracy of the one-week DASH plan for a single woman aged 20–50 years receiving SNAP benefits in an urban area in central Kentucky. Overall, under the pricing and purchasing assumptions used in this study, the plan appeared difficult to afford within the weekly food budget derived from the TFP in this regional context. Multiple serving discrepancies between the study and the plan were identified across food groups and categories. Together, these findings suggest that this meal plan may pose affordability challenges for some SNAP recipients and highlight concerns about the accuracy of serving information in a widely available federal nutrition resource.

We found that the calculated servings of fruits, vegetables, and grains based on ingredients listed in the one-week meal plan were lower than the DASH daily dietary recommendations [10]. These discrepancies may reduce dietary fiber and essential micronutrient intake, potentially diminishing the plan’s intended benefits for hypertension and CVD. They also highlight the importance of rigorously assessing publicly available standardized meal plans to ensure alignment with daily dietary and DASH recommendations. Given the launch of MyPlate serving guidelines for food groups in 2011, which preceded the release of the NHLBI one-week meal plan in 2019, more accurate serving calculations should have been feasible and accurately reported in the plan [20,23]. Therefore, this is a missed opportunity to fully align the one-week plan with the national daily dietary guidelines and DASH recommendations.

Food prices in the U.S. vary substantially by geographic region, store type (e.g., discount stores vs. stores that sell mostly local food products), seasonality, and the local food environment. Urban versus rural settings, regional supply chains, and cost of living are among the factors that can influence food prices. Seasonal variation in food prices, particularly for fresh fruits and vegetables, may also influence food prices. Prices collected at a single time point, like in this study, may not capture fluctuations throughout the year. Accordingly, the grocery cost estimates presented in this study should be interpreted as a context-specific, regional case study and may not reflect national averages or variations across other geographic settings.

Moreover, cost estimates presented in this study are based on standardized assumptions, such as the use of the lowest available regular retail prices, prioritization of store-brand products, bulk purchasing for all repeated food items in the one-week meal plan, and exclusion of temporary discounts (e.g., sales, coupons, and loyalty programs). While these assumptions were applied in this study to enhance consistency and reproducibility, actual purchasing behaviors among low-income households may differ. For example, individuals may use a variety of cost-saving approaches, such as discounts or substituting lower-cost or culturally preferred foods. Various purchasing behaviors were not included in the study methods and can influence the study’s interpretation of food affordability. Therefore, findings of this study should be interpreted as an estimate of baseline costs under standardized conditions rather than a definitive representation of all purchasing scenarios.

Additionally, geographic differences in food prices are particularly relevant when assessing the affordability of healthy diets relative to SNAP benefits, as benefit levels are determined using national averages that may not reflect local food costs. Some studies have found that TFP-based SNAP benefits may not fully align with the cost of a healthy diet in some settings [25,26]. When a national TFP threshold is used to estimate the cost of a healthy diet without accounting for geographical price variations, the affordability of that diet may be overestimated in higher-cost areas [25]. As a result, individuals living in higher-cost regions may face greater challenges in obtaining the same types of food using the same SNAP benefits. Another study found that inadequate SNAP benefits were an issue in both rural and urban settings, to varying degrees, and that substantial shortfalls in SNAP benefits persisted in both settings despite the 2023 cost-of-living adjustments [26]. Consistent with that literature, our findings suggest that this one-week plan may pose affordability challenges in this regional context. However, these results should not be interpreted as a definitive assessment of adequacy across all households, settings, or purchasing strategies. Collectively, these findings underscore the need for continued assessments of TFP, food costs across different regions, and inflation when evaluating how well SNAP benefit allotments reflect the real-world costs of heart-healthy diets.

Beyond cost-related barriers to heart-healthy eating, transportation is also a major hurdle for SNAP recipients. Commonly reported transportation constraints for SNAP participants include a lack of access to a personal vehicle, limited public transportation means, and a long distance to grocery stores, especially in rural areas [27]. In 2021, nearly 20% of SNAP participants reported a lack of transportation or a long distance to a grocery store as structural barriers to healthy food access [28]. In this study, we did not consider transportation costs to and from grocery stores, which could have a substantial impact on the overall affordability of the one-week meal plan.

Additionally, cultural relevance plays a role in the widespread adoption of the DASH eating plan [29]. The current DASH meal plan offered by NHLBI lacks cultural diversity with a heavy emphasis on Eurocentric dietary habits and food preferences. This creates a gap for those with different eating habits and food preferences due to cultural, religious, and/or other reasons. According to an integrative review, cultural relevance had a considerable impact on food choices and adherence to the DASH eating plan, particularly among Black or African American individuals who reported that some DASH-friendly foods and cuisines were unfamiliar and that available options lacked variety [29]. Overall, cost, transportation, and cultural considerations are important structural factors that can impact SNAP recipients’ ability to follow heart-healthy eating plans like DASH.

### 4.1. Strengths and Limitations

To our knowledge, this is the first study to systematically assess the affordability and serving accuracy of the NHLBI one-week plan. Cost calculations were based on retail prices from two major grocery chains commonly used by low-income households in the region, thereby improving ecological validity. Additionally, this study used the national MyPlate and American Heart Association guidelines to assess serving accuracy for food groups and categories. Verification of all study details and calculations by a registered dietitian (KO) also strengthened this study, serving as quality control and improving the validity and accuracy of interpretation.

However, this study was not without limitations. Food prices were collected from only two major grocery chains in one urban area in central Kentucky over a one-month period. Since food prices vary widely across regions, store types, seasonal product availability, and purchasing strategies, the study’s methods omitted price data from several other grocery stores across different seasons, thereby limiting the generalizability of the findings across regions. In addition, the analyzed DASH meal plan may not reflect the typical purchasing, substitution, or meal-planning strategies used by SNAP recipients, which may influence affordability in practice. Moreover, the prices of certain foods are quoted by quantity rather than weight (e.g., cantaloupe), and unknown weights limited our ability to accurately calculate the cost per serving. Due to the variability in fresh produce at grocery stores, our ability to calculate prices was affected. For example, a fresh peach in the one-week meal plan was neither in season nor available among selected vendors during the study timeframe. An additional limitation is the use of CoolConversion, a free online tool that allowed us to estimate the total number of servings and individual serving sizes, although this tool has not been validated for accuracy.

### 4.2. Future Research Directions

Future research should conduct rigorous nutrient analyses of foods listed in the NHLBI one-week plan to verify the accuracy of the nutritional values for each day and compare them with the DASH guidelines. Studies should also investigate the economic feasibility of following the DASH eating plan among SNAP participants across diverse sociodemographic groups, including older adults and men, who have different nutrient requirements than women. More research is needed to develop and/or validate weight–volume conversion tools, as the lack of a gold-standard tool currently limits the practicality and accuracy of food conversions for estimating the total number of servings, individual serving sizes, and the cost per serving.

### 4.3. Implications for Practice and Policy

This regional case study’s findings on food costs highlight the importance of aligning nutrition education resources with the most recent and reliable food cost data for a specific region. These findings also add to existing concerns about whether TFP-based SNAP benefit levels fully reflect the real-world cost of following some heart-healthy meal plans in certain contexts. Therefore, in light of regional variation in food prices, continued evaluation of the TFP methodology, including consideration of geographic variation and actual food costs when estimating SNAP benefit allotments, may be warranted.

Given the discrepancies in serving sizes for food groups and categories identified in this study, nutrition and public health professionals should continue to critically review and assess publicly available resources, including widely used federal nutrition education materials. Nutrition education resources should undergo rigorous verification and periodic updates to ensure accurate information is disseminated. In addition, future iterations of the one-week DASH meal plan should incorporate culturally appropriate and cost-effective food alternatives with exact portion sizes. Considering the potential affordability challenges and the Eurocentric focus of the one-week plan, nutrition professionals should work with SNAP participants from diverse cultural and sociodemographic backgrounds to co-create, evaluate, and validate culturally relevant, low-cost DASH eating plan resources.

For dietitians, nutrition educators, and public health practitioners, these findings underscore the need to critically assess publicly available meal plans before recommending them, especially for individuals with limited food budgets in settings where affordability may be a concern. DASH-related resources may need to be adapted using lower-cost substitutions, culturally relevant foods, and realistic meal planning strategies that reflect local food access and affordability. In this way, nutrition guidance can better align with the everyday circumstances of SNAP participants and other populations at risk of food insecurity.

## 5. Conclusions

This study systematically evaluated the affordability and serving accuracy of the publicly available NHLBI one-week DASH meal plan, identifying higher per-serving costs than the weekly food budget derived from the TFP under the assumptions used in this study as well as notable serving discrepancies for food groups and categories based on MyPlate and American Heart Association guidelines. These case study findings suggest that the one-week DASH meal plan may pose affordability challenges in this regional context and highlight the need for ongoing evaluations of how TFP-based SNAP benefit estimates align with real-world food costs across diverse geographic settings. The findings also highlight the importance of rigorous review and periodic updating of publicly available DASH resources to improve the accuracy of servings for food groups and categories and to strengthen the usefulness of these material for practice and education.

## Figures and Tables

**Table 2:** Average Weekly Food Cost based on TFP Report, Total Grocery Costs, and Adjusted Costs Per Serving for a Single Woman Aged 20–50 Years to Follow the One-Week DASH Plan in January 2025.

Average Weekly Food Cost Based on TFP Report in January 2025	Total Grocery Costs to Purchase All Food Items Listed in the One-Week Plan	Adjusted Costs Per Serving to Purchase All Food Items in the One-Week Plan
$68.40	$262.17	$82.90

**Table 3:** Total daily servings of grains, vegetables, fruits, dairy, proteins, and fats/oils compared to the one-week DASH plan and calculations in the study.

		Day 1	Day 2	Day 3	Day 4	Day 5	Day 6	Day 7
Servings of Grains	NHLBI	5.00	6.00	7.00	4.00	5.00	6.00	8.25
	Study	4.75	6.00	6.75	6.00 *	6.00 *	6.00	5.25 *
Servings of Vegetables	NHLBI	5.00	5.00	4.75	4.75	6.25	5.75	4.75
	Study	3.00 *	2.75 *	1.75 *	3.00 *	3.25 *	2.50 *	2.50 *
Servings of Fruits	NHLBI	6.00	7.00	4.00	7.00	5.00	5.00	5.00
	Study	4.50 *	4.00 *	3.00 *	3.75 *	3.50 *	3.50 *	3.50 *
Servings of Dairy	NHLBI	2.50	3.00	3.00	3.50	2.25	2.50	4.00
	Study	2.50	2.75	3.00	3.50	2.25	2.50	4.00
Servings of Proteins	NHLBI	7.50	4.50	6.25	6.00	7.75	6.75	4.50
	Study	11.00 *	7.50 *	7.00	7.50 *	9.50 *	7.50	8.00 *
Servings of Fats/Oils	NHLBI	3.50	1.50	3.00	3.00	2.00	3.50	2.50
	Study	3.00	2.50 *	4.00 *	3.50	2.75	4.50 *	2.50

* Indicates one or more serving differences between the NHLBI and study findings.

## Data Availability

The data used in the study are from the USDA Food and Nutrition Service website and the National Heart, Lung, and Blood Institute. Data files are available at the following url: https://fns-prod.azureedge.us/sites/default/files/resource-files/Cost_Of_Food_Thrifty_Food_Plan_January_2025.pdf (accessed on 26 February 2026); https://www.nhlbi.nih.gov/sites/default/files/publications/WeekOnDASH.pdf (accessed on 26 February 2026).

## Supplementary Materials

The following supporting information can be downloaded at: https://www.mdpi.com/article/10.3390/nu18091358/s1, Table S1. Serving Assignments of food items indicated in Day 6 of the one-week meal plan based on MyPlate and AHA Guidelines.

## Abbreviations

The following abbreviations are used in this manuscript:

CVD	Cardiovascular disease
DASH	Dietary Approaches to Stop Hypertension
NHLBI	National Heart, Lung, and Blood Institute
SNAP	Supplemental Nutrition Assistance Program
TFP	Thrifty Food Plan
USDA	U.S. Department of Agriculture
