# Supplementary material for: Affordability and Serving Accuracy of a Publicly Available DASH Meal Plan for Women Using SNAP Benefits
Source: Nutrients. 2026 Apr 25;18(9):1358. doi: 10.3390/nu18091358 (PMC13165375; doi:10.3390/nu18091358)
Supplement: Supplementary file 1 [file nutrients-18-01358-s001.zip › nutrients-4200380-supplementary.pdf]

Supplementary Table S1. Serving Assignments of food items indicated in Day 6 of the one-week meal plan based on MyPlate and AHA Guidelines

Meal	Ingredient	Quantity Indicated in the NHLBI Meal Plan	Number and Type of Serving based on MyPlate Guidelines
Breakfast	Low fat granola bar	1	1 serving of grain
	Medium banana	1	1 serving of fruit
	Fruit yogurt, fat free, no sugar	½ cup	0.5 serving of dairy
	Orange juice	1 cup	1 serving of fruit
	Low fat milk	1 cup	1 serving of dairy
Lunch	Turkey breast	3oz	3 servings of protein
	Whole wheat bread	2 slices	2 servings of grain
	Large leaf romaine lettuce	1	-
	Tomato	2 slices	0.25 serving of vegetable
	Mayonnaise, low fat	2 tsp	0.75 serving of fats and oils
	Dijon mustard	1 tbsp	-
	Steamed broccoli	1 cup	1 serving of vegetable
	Medium orange	1	1 serving of fruit
Dinner	Salmon fillet	3oz	3 servings of protein and 0.75 serving of fats and oils
	Scallion rice	1 cup	2 servings of grain
	Spinach	½ cup	0.25 serving of vegetable
	Canola oil	2 tsp	2 servings of fats and oils
	Silvered almond, unsalted	1 Tbsp	0.5 serving of protein
	Carrots	1 cup	1 serving of vegetable
	Whole wheat roll	1	1 serving of grain
	Soft tub margarine	1 tsp	1 serving of fats and oils
	Small cookie	1	-
Snacks	Peanuts, unsalted	2 tbsp	1 serving of protein
	Low fat milk	1 cup	1 serving of dairy
	Dried apricots	¼ cup	0.5 serving of fruit
